# Open-Cellular Co-Base and Ni-Base Superalloys Fabricated by Electron Beam Melting

**DOI:** 10.3390/ma4040782

**Published:** 2011-04-14

**Authors:** Lawrence Murr, Shujun Li, Yuxing Tian, Krista Amato, Edwin Martinez, Frank Medina

**Affiliations:** 1 Department of Metallurgical and Materials Engineering, The University of Texas at El Paso, El Paso, TX 79968, USA; E-Mails: knamato@miners.utep.edu (K.A.); emartinez21@miners.utep.edu (E.M.); 2 W. M. Keck Center for 3D Innovation, The University of Texas at El Paso, El Paso, TX 79968, USA; E-Mail: frmedina@utep.edu; 3 Shenyang National Laboratory for Materials Science, Institute of Metal Research, Chinese Academy of Sciences, Shenyang, Liaoning 11016, China; E-Mail: shjli@imr.ac.cn (S.L.)

**Keywords:** superalloys, mesh and foam structures, dynamic stiffness, electron beam melting, microstructures

## Abstract

Reticulated mesh samples of Co-29Cr-6Mo alloy and Ni-21Cr-9Mo-4Nb alloy (625) and stochastic foam samples of Co-29Cr-6Mo alloy fabricated by electron beam melting were characterized by optical metallography, and the dynamic stiffness (Young’s modulus) was measured by resonant frequency analysis. The relative stiffness (E/E_s_) *versus* relative density (ρ/ρ_s_) plotted on a log-log basis resulted in a fitted straight line with a slope n ≅ 2, consistent with that for ideal open cellular materials.

## 1. Introduction

Open-cellular metals, including reticulated mesh structures and stochastic foams (also technically reticular structures) represent a wide class of novel engineering materials. They offer opportunities which include a variety of ultra-light-weight structure applications, thermal management and especially heat transfer systems, high energy absorption for blast and crash systems, and energy storage devices among others [1,2,3,4,5,6,7,8,9,10]. Ideally, the complex lattice of porous matter can be subdivided into smaller, single elements such as rods, struts, or connecting ligaments, as well as shells or sheets. Ideally, these structures can be considered as some form of regular-faced, convex polyhedral [11] which characterize the void spaces or void volume. Numerous process routes have been developed to make cellular metal structures, including vapor growth forms, electro-deposition from aqueous solutions onto expendable forms, liquid casting and microcasting, and powder methods (including hollow and solid spheres) [12,13,14,15]. While aluminum and aluminum alloys have been commercially available for decades, other technically important metals and alloys have been difficult to produce especially with small strut or ligament dimensions in the range of hundreds of microns. Recently, however, several forms of additive manufacturing, utilizing either laser or electron beam melting of metal or alloy powders, have allowed for the fabrication of small dimension mesh or foam structures [16,17,18,19,20].

In ideal cellular materials, unlike their solid (fully dense) forms, plastic yielding and collapse occur simultaneously [2] while some cellular elements allow bending as a consequence of high local stresses causing the system to be compliant and to have a low yield strength. Stiff and high strength elements will stretch without bending. Consequently, open-cellular structures are usually susceptible to bending, causing their stiffness (or Young’s modulus) to be relatively low. However, stiffness (or elastic modulus, E) has been shown to be subject to a general scaling law of the form [2]:

E/E_o_ ≅ (ρ/ρ_o_)^n^ (1)

where E and ρ are the Young’s modulus and density for the open-cellular structure, and E_o_ and ρ_o_ are the same properties for the same solid (fully dense) structure. While values of n have ranged from ~1.8 to 2.2 [2], n = 2 is the rule of thumb. The ratios E/E_o_ and ρ/ρ_o_ are also referred to as the relative stiffness and relative density, respectively. For many structural applications, stiffness and strength (yield strength, σ) at low weight or density are important. Common measures of axial stiffness and strength include so-called specific stiffness (E/ρ) and specific strength (σ/ρ). In this study, we have fabricated Co-base (Co-26Cr-6Mo) superalloy mesh and foam structures and Ni-base (alloy 625) superalloy mesh structures using electron beam melting (EBM). We have characterized these structures and corresponding microstructures using optical and scanning electron microscopy, and measured their densities, and corresponding dynamic stiffnesses using resonant frequency analysis.

## 2. Experimental

This research program utilized pre-alloyed powders of a Co-base alloy having a composition of 29Cr, 6Mo, 0.7Si, 0.5Mn, 0.25Ni, 0.22 C and the balance Co (in weight percent), and Ni-base alloy 625 having a composition of 21Cr, 9Mo, 4Nb, and the balance Ni (in weight percent). The average, spherical powder particle sizes were 40 µm and 22 µm for the Co-base and Ni-base alloys, respectively. The EBM system, an Arcam A2, and the build parameters, have been described in detail elsewhere [19,20].

**Figure 1:**
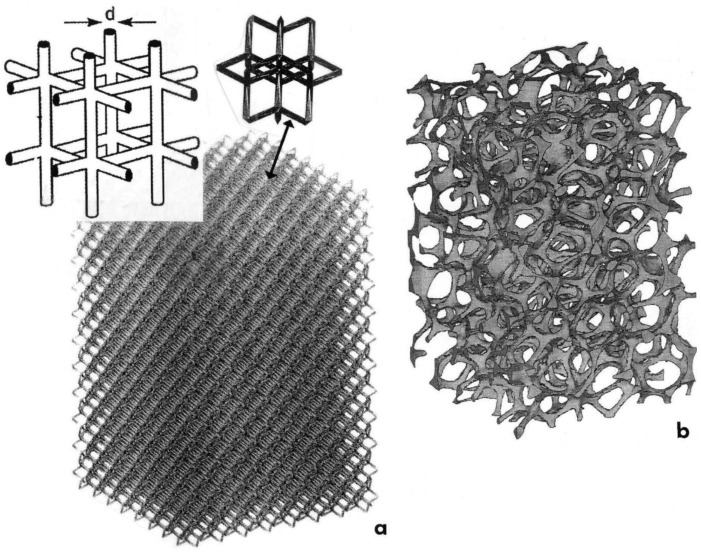
CAD-models for EBM fabrication of open-cellular structures. (**a**) Dode-thin (Materialise^™^) software element (arrow) and model. A simple strut structure having strut diameter, d, is shown inserted upper left. (**b**) Cellular foam model based on microCT scan element.

The mesh structure fabrication utilized a materialize/magics software package to construct CAD models from selected structure generators described and illustrated previously [19]. Figure 1(a) illustrates this feature. The model insert shows a simple strut in the upper left with the modified dode-thin build element shown by the arrow [19]. The foam structure fabrication utilized a foam structure generator constructed from a CT scan of a standard aluminum foam, as illustrated in the CAD model shown in Figure 1(b) and described in more detail by Murr *et al.* [19]. These models directed the selected melting of additive layers to build the characteristic components. In addition to the open-cellular structures, solid cylindrical specimens were also fabricated of each alloy in order to be able to compare solid microstructures and properties.

The dynamic stiffnesses (E) were measured using a resonant frequency (f_r_) analyzer (IMCE-HTVP 1750-C), where E ∝ f_r_^2^. Specimen shapes, for measurement, were limited in dimensions to optimize f_r_, and were, in some cases, carefully cut from larger fabricated specimens. However, the apparent moduli and strength of test samples depends on the ratio of the specimen size to the cell (pore) size as well as gripping and loading. Consequently, specimens were designed to satisfy the general requirements for metal foams discussed by Ashby *et al*. [4] where the height/width (or thickness) >1.5; height >7 times the cell size or largest pore or channel size.

## 3. Results and Discussion

**Figure 2:**
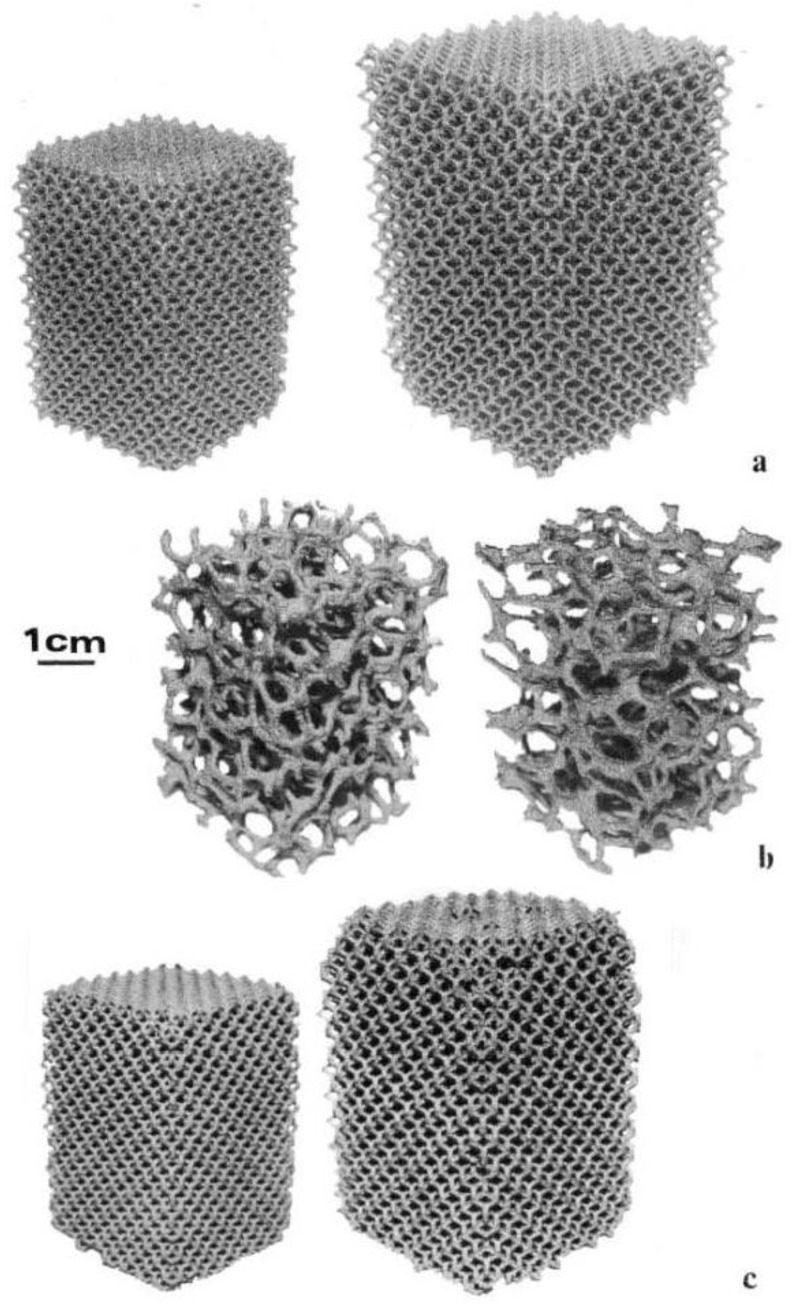
Examples of electron beam melting (EBM) fabricated open-cellular structures. (**a**) Co-Cr-Mo mesh samples having densities of 0.98 g/cm^3^ (left) and 1.25 g/cm^3^ (right); (**b**) Co-Cr-Mo foam samples having densities of 0.66 g/cm^3^ (left) and 0.69 g/cm^3^ (right); (**c**) Ni-base alloy 625 mesh samples having densities of 1.37 g/cm^3^ (left) and 1.80 g/cm^3^ (right).

**Figure 3:**
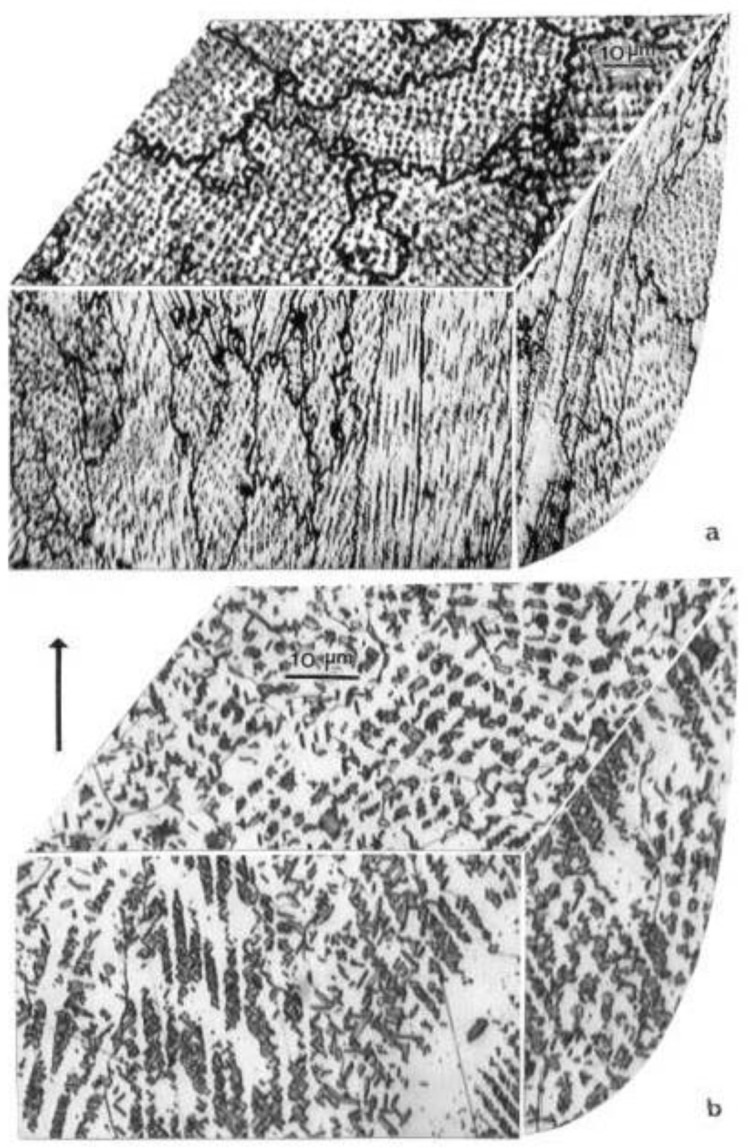
3D microstructure composition views for (**a**) Co-Cr-Mo and (**b**) Ni-Cr-Mo-Nb mesh interiors oriented with respect to the build direction (arrow).

Figure 2 shows some examples of the EBM-fabricated Co-base and Ni-base mesh and foam samples. Figure 3 compares examples of the mesh strut 3D microstructures for these alloys (Figure 2). The details of these microstructures or microstructural architectures for the Co-base and Ni-base alloys have been described in detail for solid EBM components by Gaytan *et al.* [20] and Murr *et al.* [21], respectively. Comparing Figure 3(a) and (b), it is observed that precipitate columns are formed parallel to the build direction as a consequence of the electron beam melt scan parameters and scan geometry [19,20]. This creates micron-size melt pools which act as additive, directional solidification regimes thermodynamically similar to classical directional solidification [22,23] which has produced eutectic unidirectional architectures similar to the columnar precipitate architectures shown in Figure 3 [24,25]. These features for EBM-produced oriented or directional microstructures have been described by Thijs *et al.* [26] for Ti-6Al-4V, by Gaytan *et al.* [20] for the Co-Cr-Mo alloy, and by Strondl *et al.* [27] for a Ni-base alloy (Inconel 718) similar to those shown in Figure 3(b). The 3D composition in Figure 3(a) shows Cr_23_C_6_ precipitates composing the columnar architectures in the Co-Cr-Mo alloy which has been discussed in more detail by Gaytan *et al.* [20]. Figure 3(b) shows bct Ni_3_Nb precipitate platelets coincident with the alloy 625 fcc {111} planes described in detail by Murr *et al.* [21] while Figure 3(a) shows only the mesh strut microstructures. The foam ligament (Figure 2(b)) microstructures are identical.

The precipitate columns are more irregular or erratic in the open-cellular structures than solid EBM components [20,21] because the cooling rates are different. The additive-layer fabrication process is itself much less uniform in contrast to more traditional solidification, especially directional solidification characterized by a solid/liquid solidification boundary [23].

Table 1 reproduces the stiffness (E) and corresponding density (ρ) measurements along with the relative stiffness (E/E_o_) and relative density (ρ/ρ_o_) values for the Co-base alloy mesh and foam samples and Ni-base alloy mesh samples. Table 1 also lists the Vickers microindentation hardness averages for the corresponding mesh and foam samples, along with the specific stiffness (E/ρ) and specific strength (σ/ρ) values; where the yield strength (stress), σ, was estimated to be HV/3, which is usually an over estimate of solid yield strength. For example, tensile yield stress (0.2% engineering offset) measurements for the as-fabricated Co-base alloy and Ni-base alloy cylindrical specimens were 0.5 and 0.4 GPa, respectively. Corresponding Vickers microindentation hardness (HV) measurements were 2.8 GPa and 4.5 GPa, respectively. It can be observed in Table 1 that the highest specific stiffness for these open-cellular structures (2.00 GPa/(g/cm^3^)) was recorded for the Co-base alloy mesh with the highest density of 1.85 g/cm^3^. This is in contrast to a specific stiffness of 25 GPa/(g/cm^3^) for both the Co-base and Ni-base alloys. Since E_o_ = 210 GPa and ρ_o_ = 8.44 g/cm^3^ for both. In contrast, the common specific strength was measured to be ~0.20 and 0.16 GPa/(g/cm^3^) for the Co-base and Ni-base alloys, respectively, while the highest specific strength for the open-cellular structures indicated in Table 1 is observed to occur for the 0.63 g/cm^3^ Co-base alloy foam: ~2.3 GPa/(g/cm^3^).

**Table 1:** Density, Modulus, Hardness Measurements and Related Values for Co-Base and Ni-Base Alloy Mesh and Foam Structures.

MATERIAL	ρ^a^ (g/cm^3^)	Pore^h^ Density (ppi)	ρ/ρ_o_^b^	E^c^ (GPa)	E/E_o_^d^	E/ρ^e^	HV^f^ (GPa)	σ/ρ^g^
Co-base mesh	0.98	14	0.12	0.49	2.3	0.50	5.2	1.8
Co-base mesh	1.25	17	0.15	1.16	5.5	0.93	-	1.4
Co-base mesh	1.85	25	0.22	3.68	17.5	2.00	-	0.9
Co-base foam	0.63	4	0.075	0.25	1.2	0.40	4.5	2.3
Co-base foam	0.66	5	0.078	0.28	1.3	0.42	-	2.3
Co-base foam	0.69	7	0.080	0.35	1.7	0.50	-	2.2
Co-base foam	0.76	9	0.090	0.70	3.3	0.92	-	2.0
Ni-base mesh	1.37	14	0.16	0.76	3.6	0.55	2.9	0.7
Ni-base mesh	1.80	17	0.21	1.68	8.0	0.93	-	0.5
Ni-base mesh	2.60	22	0.31	4.17	19.9	1.60	-	0.4

^a^ Density; ^b^ Relative density; ρ_o_ = 8.44 g/cm^3^; ^c^ Stiffness (Young’s Modulus); ^d^ Relative Density; E_o_ = 210 GPa; ^e^ Specific Stiffness (units of GPa-cm^3^/g); ^f^ Vicker’s microindentation hardness; ^g^ Specific Strength; σ ≅ HV/3 (yield stress) (units of GPa-cm^3^/g); ^h^ Pore density is the number of pores measured in a linear inch: pores per inch (ppi) (averaged across the flat face).

## 4. Conclusions

**Figure 4:**
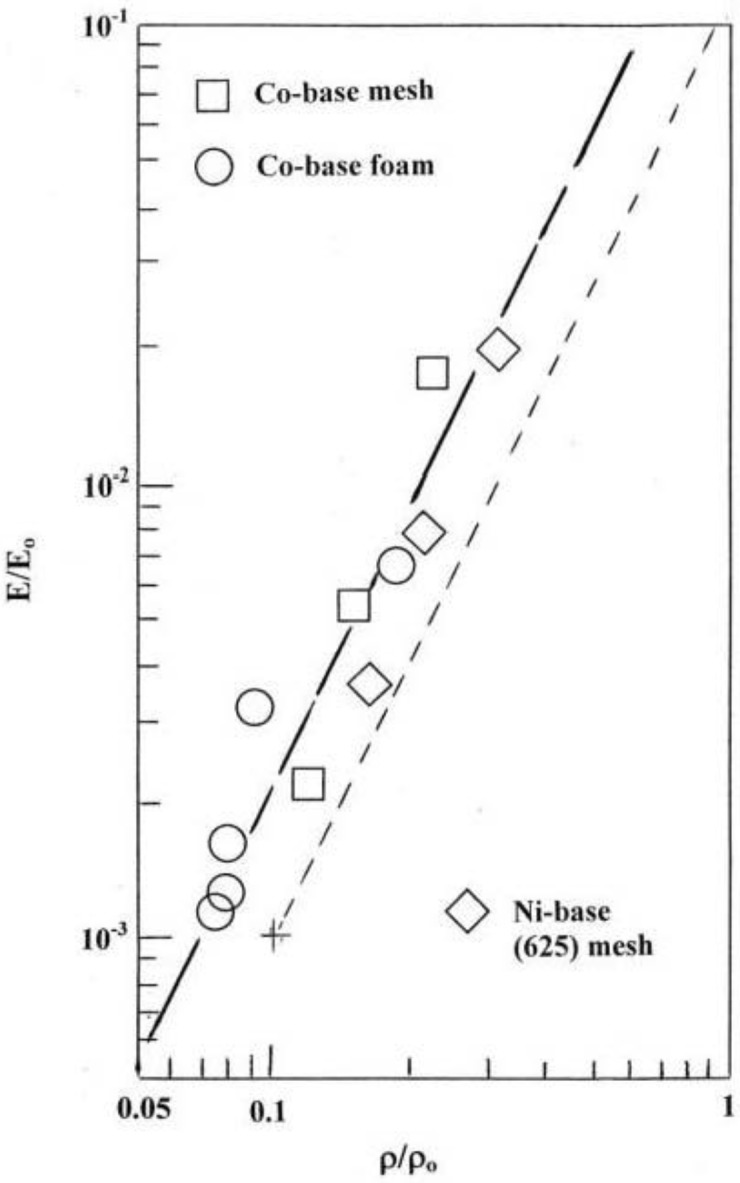
Relative stiffness (E/E_o_) *versus* relative density (ρ/ρ_o_) for open cellular Co-Cr-Mo and Ni-base alloy 625 (from Table 1). The dashed line, parallel to the fitted data line, corresponds to a slope of ~2.

Plotting the relative stiffness *versus* relative density in a log-log graph as shown in Figure 4 for the Co-base and Ni-base alloy open-cellular structures in Table 1, illustrates that these alloy structures can be uniformly fitted to a straight line having a slope, n, in Equation (1), of ~2 (dashed parallel adjusted slope), corresponding to an ideal foam or open-cellular metal. The value of n could be quantified as 2.2 which is not in contrast to a slope of ~2.3 for fitted Ti-6Al-4V mesh and foam structures fabricated by EBM in the work of Murr *et al.* [19].
